# Unveiling Mesenchymal Stromal Cells’ Organizing Function in Regeneration

**DOI:** 10.3390/ijms20040823

**Published:** 2019-02-14

**Authors:** Peter P. Nimiritsky, Roman Yu. Eremichev, Natalya A. Alexandrushkina, Anastasia Yu. Efimenko, Vsevolod A. Tkachuk, Pavel I. Makarevich

**Affiliations:** 1 Institute for Regenerative Medicine, Medical research and education center, Lomonosov Moscow State University, Moscow 119192, Russia; nimiritsky@gmail.com (P.P.N.); romaneremichev@gmail.com (R.Y.E.); n.alexandrushkina@gmail.com (N.A.A.); aefimenko@mc.msu.ru (A.Y.E.); tkachuk@fbm.msu.ru (V.A.T.); 2 Faculty of Medicine, Lomonosov Moscow State University, Moscow 119192, Russia; 3 Laboratory of Molecular Endocrinology, National medical research center of cardiology, Moscow 121552, Russia

**Keywords:** stem cell, stromal cell, mesenchymal stromal cell, regeneration, cell sheet, cell delivery

## Abstract

Regeneration is a fundamental process attributed to the functions of adult stem cells. In the last decades, delivery of suspended adult stem cells is widely adopted in regenerative medicine as a leading means of cell therapy. However, adult stem cells cannot complete the task of human body regeneration effectively by themselves as far as they need a receptive microenvironment (the niche) to engraft and perform properly. Understanding the mechanisms underlying mammalian regeneration leads us to an assumption that improved outcomes of cell therapy require a specific microenvironment that is generated in damaged areas prior to stem cell delivery. To a certain extent, it may be achieved by the delivery of mesenchymal stromal cells (MSCs), not in dispersed form, but rather in self-organized cell sheets (CS) – tissue-like structures comprised of viable cells and microenvironment components: extracellular matrix and soluble factors deposited in the matrix. In this review, we highlight the potential role of MSCs as regeneration organizers and speculate that this function emerges in CS. This concept shifts our understanding of the therapeutic mechanism underlying a widely known CS-based delivery method for regenerative medicine.

## 1. The Challenge for Stem Cell Therapy: Importance of Microenvironment

In the last decades, stem cell therapy has moved from using fetal and embryonic material to adult stem cell applications pursuing the goal of structural and functional restoration of damaged tissue [1]. Adult stem cells localized in the majority of organs drive their physiological renewal and recovery after damage or disease [2]. This natural mechanism of human body regeneration provides a promising tool for regenerative medicine and resolves the ethical problems related to the use of fetal material, as well as major safety concerns connected with the use of undifferentiated and/or potentially teratogenic cells [3,4].

Despite the initial promise of stem cell therapy, it showed limited efficacy in numerous clinical trials [5], thus, placing doubt on the initial concept portraying adult stem cells as regenerative “commando engineers” that can engraft, differentiate, and rebuild fully functional tissue once delivered viable and in sufficient amounts [6]. Eventually, accumulated data led to the conclusion that most delivered stem cells fail to integrate or give rise to new tissue elements, but rather act as “bystanders” releasing paracrine factors, and disappear within several weeks [7,8,9].

Discussing the reasons for disappointing results of stem cell therapies, we may question what can be improved to overcome hurdles that stand in the way of these promising approaches. Here we turn to the role of microenvironment required to maintain finely tuned physiological functions of stem cells [10]. This concept is known since Raymond Schofield’s seminal work suggesting that stem cells are “non-autonomous” and depend on the microenvironment known as the *niche* [11]. The niche controls, directs, and supports balanced stages of stem cell’s life cycle and includes other cells (stromal, parenchymal, vascular, etc.), extracellular matrix (ECM), soluble proteins and peptides, extracellular vesicles, small molecules, and chemical factors (pH, oxygen pressure, etc.) [12]. 

The importance of the microenvironment for success of stem cell-driven body repair has been supported by investigations of regeneration in species with ability to rebuild body parts or even whole organism after injury (e.g., planarians, starfish, axolotl). In these creatures, “separated” adult stem cells (neoblasts) themselves lack the ability to drive full-scale regeneration and require certain amount of tissue to perform properly [13]. 

Human adult stem cells administered in suspension to sites of damaged are deprived of stimuli required for their regenerative potential to unfold. They lack appropriate intercellular contacts, nutrition, and regulatory signals. Under such unfavorable conditions, formation of mature tissue elements from transplanted stem cells is an extremely rare event, hardly providing significant therapeutic impact [14,15]. 

In this paper, we summarize our vision on the problems that stand in the way of successful application of stem cell therapies. We focus on a cornerstone role of the microenvironment formed during regeneration and the contribution of mesenchymal stromal cells (MSCs) to this process. We suggest that tissue engineered constructs known as cell sheets (CS) present a feasible tool to unfold the potential of MSCs as organizers of regeneration. 

## 2. Shifting the Focus to the Microenvironment: Feeder Needed!

To illustrate the possible reasons for stem cell therapy failure and to support our emphasis on the importance of a receptive microenvironment for success of this therapeutic approach, we shall start with a metaphor to compare mammalian tissue regeneration with repopulation of an ecosystem after a natural catastrophe.

**Figure 1:**
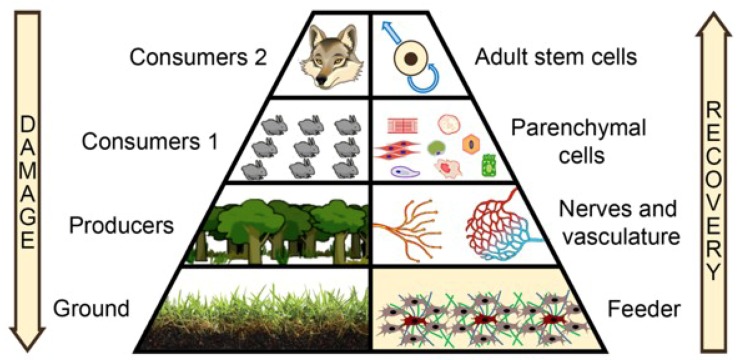
Critical role of feeders in the sequential regeneration of the human body. Both in ecosystems and the human body, “ground” and “feeder” levels, respectively, are generated by the most adaptive and universal inhabitants. They have critical importance as they provide the foundation for subsequent interactions between elements and the system structure recovery (see text for detail).

It is well known that the structure of ecological systems has a hierarchy based on food chains. It is typically portrayed as a pyramid reflecting dependency of high-order consumers on lower-order and down to the ground level of energy-absorbing producers—plants and microorganisms (left part of Figure 1). 

After a disaster, the hierarchy of an ecosystem and its structure recovers stage by stage from the foundation, starting with the most adaptive and viable species—the producers. When they generate a necessary trophic substrate, then more demanding species can enter this system and populate it. Gradually the species diversity of the entire ecosystem increases and populations begin to interact, controlling each other until the hierarchy of the ecosystem is restored and full recovery occurs [16]. However, the cornerstone of this process is—and it should be emphasized—the formation of a “ground level” of producers that is required for other species to survive [17].

This metaphor was used to show why therapies using stem cells may fail. In the acute phase of response to injury, we deliver them to a microenvironment lacking necessary elements and regulatory framework that existed before damage. We speculate that the stages of an ecosystem recovery might portray the regeneration in humans and illustrate that stem cells are capable of unveiling their potential only when an adequate microenvironment is generated prior to that. Indeed, after damaged area has been cleansed by inflammatory cells, it becomes “ground zero” with disrupted structure of the tissue down to the molecular level. Under such conditions, the stem cells fail to rebuild the tissue as they depend on other elements—ECM, soluble factors, endothelium, stromal cells, and neural terminals that existed prior to damage [10,11,12]. 

In ecological terms, this may be described as a high-order consumer entering the vast field of ashes after a forest fire before the lower levels are re-populated. Failing to find food and mate, this higher-order consumer will eventually leave the unsuitable habitat. Following this idea, the basal layer of the right side of the pyramid, the *feeder* (right part of Figure 1), becomes of paramount importance for microenvironment rebuilding to involve specific cell types (including stem cells), which is similar to the ecological dogma that producers’ presence is obligatory prior to appearance of consumer populations. 

We define the *feeder* as a transient heterogenous structure formed in vivo at an early stage of regeneration to re-create the disrupted components of the microenvironment, which allows other cell types to perform properly during recovery of structure and function. In our concept, this putative feeder may contain stromal, immune, and probably vascular cells and intercellular components of the microenvironment: ECM and soluble factors predominantly anchored in the matrix to render paracrine effects and create gradients of stimuli, mapping further the stages of regeneration. 

A similar requirement for feeders has been described in zoology prior to the formation of blastema after the amputation of a limb in the salamander. Immediately after injury, a layer of epidermal cells covers the wound and becomes an *apical epithelial cap*, providing ground for the attraction of fibroblasts and other cells that dedifferentiate to form the blastema and eventually regrow the limb [18].

Nevertheless, even in widely acknowledged cases of full regeneration, there is an extremely important aspect of “missing tissue” or “tissue absence” sense, which is crucial for discerning between damage that requires *healing* and the loss of body parts or even decapitation that requires *regeneration*. In planarians, this is mediated by the follistatin–activin axis [19]. Thus, not only damage itself but the ability of an organism to sense “tissue absence” is crucial for the activation of regeneration. Mammalian tissues show follistatin and activin expression with the highest levels observed in skin and skeletal muscle. However, it remains unknown why, despite having all the components of this axis available, the discernment between damage and tissue loss in humans is not possible [20,21].

An encouraging example of whole organ recapitulation from delivered stem cells in humans is bone marrow transplantation [22]. However, in this procedure after the myeloablative stage, the stromal component of bone marrow remains relatively intact, providing a functional niche for the transplanted hematopoietic cells. This supports the idea that a receptive microenvironment is critical for regeneration, allowing stem cells to rebuild tissue, but not work as secretome-producing “bystanders” [14,15]. 

A typical example of a non-receptive microenvironment that can be formed after damage is a scar, which is an avascular bulk of ECM packed to hold the tissues together and barely undergoing resolution over time. Stem cell therapies have shown little to no effect in the stimulation of fibrosis resolution, yet in some disorders, such as cystic fibrosis or idiopathic pulmonary fibrosis, they have been shown to attenuate disease progression [23]. Nevertheless, these effects were once again generally attributed to cells’ paracrine function rather than engraftment and tissue restoration [8]. 

## 3. Rebuilding the Feeder: Focus on Mesenchymal Stromal Cells (MSCs)

Thus, we may conclude that stem cell therapies do not achieve their goal until a receptive microenvironment is formed. Due to a defined order of events during regeneration after injury, we suggest focusing not only on tissue-specific stem cells but also on the basal layer for which we provide the term *feeder* (in particular, similar to the cell culture technique used to support stem cells and other cell types). Below we speculate on the participation of MSCs in its formation in vivo and address their biological and physiological functions that support this point.

Among the numerous cell types of the human body, MSCs remain a prominent candidate for therapeutic use and in our concept, they comprise an important (yet not the only) component of the putative feeder. In the human body, MSCs are involved in maintaining the structure and homeostasis [24], comprising a ubiquitous stromal component in the majority of organs. In a quiescent state, they are localized in perivascular compartments supporting vascular permeability, secrete growth factors, and support ECM production and turnover. These functions are important for the interactions with local tissue progenitors and stem cells to retain organ architecture by maintaining gradients of stimuli. In intestinal crypt niches, MSCs create a gradient of bone morphogenetic proteins (BMPs) that control resident stem cell fate and differentiation [25]. However, this tissue function of MSCs is not limited to stem cell niches. In intact organs, they also participate in the formation of microenvironments for all cells that are located nearby [26]. 

After damage, MSCs migrate to the lesion site where they actively proliferate [27]. Their ability to survive under stress results in the excellent cultural properties of isolated MSCs. Upon completion of the acute phase of inflammation, MSCs attenuate the intensity of residual inflammation. They secrete a wide range of anti-inflammatory factors (IL-10, TSG-6, PGE_2_) and, thus, switch the microenvironment to a pro-regenerative state, reducing oxidative stress and toxic compound production by immune cells [28]. Further, MSCs produce ECM proteins and factors of matrix turnover (matrix metalloproteases, etc.), as well as cytokines and growth factors to attract and support other cell types (endothelial, tissue-specific progenitors, etc.) [28,29,30]. 

This guided us to investigate the composition of MSC secretome, and our group, as well as others, found that the physiological potency of the secretome is sufficient to trigger events crucial for regeneration [31,32,33]. It activates the proliferation and survival of different cells under stress, protects tissue from excessive fibrosis, induces angiogenesis and nerve growth, and regulates the immune response, etc., leading to the idea of secretome therapeutic application known as “cell-free cell therapy” [34,35,36]. Our further efforts were focused on the functionalization of MSC secretome by viral delivery of growth factor genes or pre-treatment by recombinant proteins. We have shown that this results in a significant shift of MSC functional activity and improvement of therapeutic effect [37,38,39]. However, we did not find an increase in engraftment after functionalization, leading us to generally support the conclusion that MSC therapies succeed in delivering secretome produced by stem cells over time rather than replenish native tissue elements by their differentiation. In brief these features are summarized in Table 1 below.

Thus, after injury, damage-activated MSCs mitigate the inflammatory response and produce basic elements of the putative *feeder*: ECM and soluble trophic factors to attract other cells, to interact with them and form a basis for regeneration. 

Nevertheless, one may wonder why MSCs delivered by injection fail to form interactions between themselves and drive regeneration. An available answer is that a negative influence of ECM loss and contact disruption due to harvesting from a monolayer followed by injection makes MSCs unable to perform their functions [49]. It is predominantly related to stress due to loss of temporary microenvironment that existed in vitro; namely, the adhesive surface, ECM, and soluble factors of the culture medium. Certain reports claim that after injection, up to 70% of MSCs undergo cell death within 48–72 h after delivery [43].

Thus, MSC therapy is limited to bystander effects similar to other stem cell therapies. However, MSCs are able to produce basic components of their own microenvironment: ECM and pro-survival soluble factors. Moreover, they are able to organize this microenvironment and generate a tissue-like structure in vitro; namely, a cell sheet (CS). The latter is generally used to facilitate cell delivery and transplant MSCs in an organized manner resolving the problem of dispersed cells’ inability to survive without an appropriate microenvironment. Below we shall provide our point of view and data that support CS-based MSC delivery as a more physiological approach, potentially providing ground for effective human regeneration.

## 4. Cell Sheet Technology: Basics and Application

Cell sheets are scaffold-free tissue-like constructs consisting of living cells, ECM, and soluble factors accumulated during routine culture. The formation of CS varies in time (3–14 days), depends on the ECM and soluble factor production, cell proliferation, and their ability to integrate into a multilayered construct that detaches from thermo-responsive coatings [50] or after treatment by chelating agents and/or mechanical peeling [51]. 

This technology quickly found application in many areas of regenerative medicine, primarily due to the advantages over direct injection. Delivery by CS is a method that delivers cells along with the microenvironment that formed in vitro. Many preclinical and clinical studies have reported on CS efficacy to treat diseases of the bones, cartilage [52,53], skin, urinary bladder, heart, blood vessels, esophagus, and cornea, etc. [54]. To better understand the details and therapeutic promise of CS technology in regenerative medicine, we direct the reader to a number of recent seminal reviews [55,56,57,58,59].

Experimental studies to treat wounds, limb ischemia [60], nerve damage [61], and myocardial infarction [62] have been published by our group during the last decade. Besides the delivery of primary MSC sheets, we have studied endothelial cells, cardiac stem cells [51], and viral modification of CS to enhance therapeutic outcomes [60,61,63] and our data along with published works of other researchers allows two general conclusions regarding CS as a delivery platform for MSC therapy:

### 4.1. Delivery of MSCs Assembled in CS Results in a Strong and Reproducible Increase of Efficacy in Models of Tissue Damage

Comparison of efficacy after delivery of equivalent amounts of MSCs by injection or by CS transplantation has shown that sheets are superior to injected cells. In our studies, CS have performed better than suspended MSCs in the treatment of ischemia, nerve repair [60,61,64], deep wound healing [65], and pressure ulcer. Recent papers describing the use of CS for liver regeneration [66] or bone healing [67] do not report direct “head to head” comparison of outcomes after CS or suspended delivery, yet most of them report long-term survival of transplanted cells suggesting that it mediates therapeutic outcome.

### 4.2. CS from MSCs Engraft, Show Vascularization and Facilitate MSC Survival and Proliferation 

This point was illustrated in one of our works using subcutaneous implantation of labeled MSC sheets in mice indicating CD31-positive capillaries and larger caliber blood vessels in CS mass, as well as Ki-67-positive MSCs detected 2 weeks after transplantation [60]. Our results were supported by data from other groups using MSC sheets [68,69,70] and by our own data using other cell types, namely cardiac stem cell sheets transplanted to treat myocardial infarction in a rat model [51,62]. 

The points mentioned above support our suggestion that assembly of CS from MSCs facilitates *engraftment* and, thus, the delivery of a stromal part of the suggested feeder that may become a putative basis for further regeneration. However, to do this, MSCs must interact with other cell types and integrate into an organotypic structure. The potential mechanism for that is the generation of stimuli gradients, which will be explained further.

## 5. Role of MSC in Structure Organization in Development and Regeneration

Early stages of human development are driven by embryonic stem cells’ (ESCs) ability to interconnect and integrate into a community [71]. Crosstalk between ESCs dynamically changes form and structure, resulting in embryogenesis. Mesenchyme appears during the gastrulation phase and gets involved in subsequent organogenesis. Interactions of mesenchymal cells with epithelial cells are crucial in the development of teeth [72], lungs [73], pancreas [74], heart [75], kidney [76,77], and liver [78].

A critical aspect of the mentioned processes in development is the formation of stimuli gradients including those produced by mesenchymal cells [79]. Soluble factors, ECM, and cell-to-cell contacts have been postulated to participate in accurate organogenesis of many organs and the role of mesenchymal cells acting as the main structure organizers is well established as well [80,81]. 

In the postnatal period, MSCs (despite losing many traits they featured in development) may retain the organizing ability that they use for regeneration, and the molecular basis of this ability is the generation of stimuli gradients once the regenerative program is activated by injury. This supports our concept of the role of MSCs in the generation of the putative feeder and explains how they can facilitate and “map” the next stages of regeneration.

## 6. MSCs as Organizers of Other Cell Types: ex vivo Evidence

MSCs’ ability to support and organize other cells in vitro has been reported previously for epithelial cells from embryonic salivary gland. In this study, MSCs, but not conventionally used feeders (NIH/3T3 fibroblasts), supported self-assembly of primary embryonic epithelial cells and subsequent branching with bud formation in 3D culture resembling mesenchyme function in development [82].

One of the strongest pieces of evidence of MSCs’ ability to facilitate organogenesis in vitro is work by Takebe et al. [83] describing MSC-driven condensation of iPS-derived progenitors to form bud-shaped organoids. The spectrum of organoids that can be successfully assembled under the influence of MSCs in vitro includes liver, intestine, lung, kidney, heart, and brain. Finally, endothelium-containing pancreatic and renal buds derived under the influence of MSCs were transplanted into animals where they rapidly underwent vascularization and self-organized into functional, tissue-specific structures. 

The organization of such complex organ(oid)s as a pancreas or kidney should employ the same principles as in organogenesis, relying on focal stimuli provided by MSCs to organize other cell types properly. At this point, our recent data describing heterogeneity in human MSC-derived CS provides a new outlook on this delivery mode and its potential impact on regeneration [84,85].

**Figure 2:**
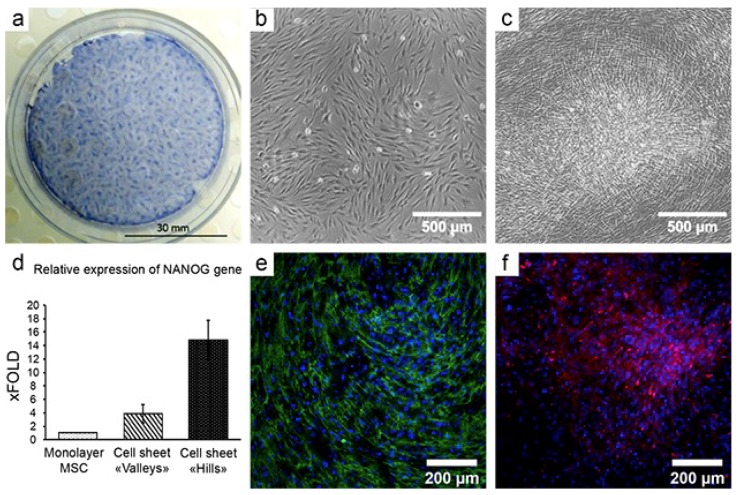
Self-organization of mesenchymal stromal cells (MSCs) in cell sheets (CS) results in the formation of a heterogeneous microenvironment. (**a**). Heterogeneous distribution of MSCs in CS at day 12 of culture; hematoxylin staining; b–c. Microphotographs taken in the same field of view of MSC culture at day 1 (**b**) and day 12 (**c**). Formation of a high-density “hill” is observed as a result of MSC self-organization in CS; time-lapse microscopy, phase-contrast. (**d**). MSC subpopulations were picked by laser microdissection. MSCs in the “hills” show increased expression of stemness-associated factor (NANOG); real-time qPCR (*n* = 3); data expressed as mean ± standard deviation. e–f. MSCs in CS deposit EDA-fibronectin in regular network of fibers (green, panel **e**) while laminin (red, panel **f**) is deposited primarily in “hills” but not in “valleys” creating foci of extracellular matrix deposition in CS (**f**); immunofluorescent microscopy, nuclei are stained by DAPI (blue); from Nimiritsky et al. [84].

In our study, a CS from human adipose-derived MSCs showed significant morphological heterogeneity with two distinct compartments of high and low density of cells (“hills” and “valleys,” respectively – see Figure 2a–c). High-density regions were formed as a result of MSC proliferation cycles followed by coordinated contraction and migration of cells (see Supplementary video 1), which resembles a previously described contraction phenomenon that accompanies MSC-driven self-organization. 

For details of the experimental procedures, please refer to the Supplementary Materials and Methods online (Table S1 and Figure S1).

Mesenchyme-mediated guidance in development relies on ECM differential distribution that has crucial importance as it facilitates the attraction and retention of different cell types [80]. We found that in MSC sheets, two ECM proteins—EDA-fibronectin and laminin—were distributed unevenly with a higher density in “hills” and a lower density in “valleys” (Figure 2e,f). Condensed laminin deposition was detectable only in “hills” of CS and in the monolayer of MSCs, no acquirable signal was detected. Laminin is known to mediate the interaction of mesenchymal and epithelial cells leading to the assumption that heterogeneity of it in CS reflects the formation of guiding ECM stimuli that can orchestrate the organization of endothelial, epithelial, and mature stromal cells on this in vitro formed feeder.

Differential morphology and ECM deposition in CS suggest that different MSC subtypes are retained in them. Therefore, we used laser microdissection to separate the “hills” and “valleys” at day 12 to evaluate relative expression of stemness-related gene *NANOG* in MSCs from different microenvironments. 

We compared laser-dissected “hills” vs. remaining “valley” portions of CS and found a significantly (up to 15-fold) increased *NANOG* expression compared to “valleys” of the same CS (Figure 2d). This suggested that heterogeneity of CS results in either the sorting of different cells to different compartments of the construct or local shifts of stemness in situ resembling the “mapping” function of mesenchyme known in development and suggests that MSC sheets actually can be considered as a guiding construct rather than just a delivery tool.

**Figure 3:**
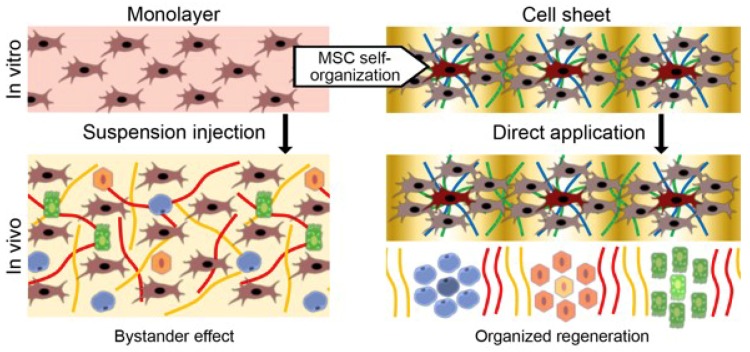
Self-organization of MSCs as a basis of their organizing function in regeneration. Conventionally used injection of MSCs cultured in monolayer (left part) is limited to paracrine (bystander) effects of delivered cells. Self-organization during CS formation results in retention of the microenvironment with the formation of stimuli gradients that organize regeneration after delivery (right part).

Previously, MSCs have been successfully used to generate pre-vascularized structures. Endothelial cells spontaneously organized into tubes during co-culture with MSCs undergoing sheet formation [82,86]. This provided a method for the fabrication of tissue-like 3D structures by stacking pre-vascularized CS together without loss of nutrition inside the construct. Nevertheless, our data suggest more attention be given to the potential use of MSC-based CS as an effective driver of organoid or tissue self-assembly as it possesses an important “mapping” trait that is required for organization of other cell types by gradients of stimuli (Figure 3).

Previous data obtained by our group and other authors using single cell analysis found numerous subpopulations in primary MSCs with varying sensitivity to hormones, differentiation ability, and signaling [87,88,89]. However, the origin of stimuli gradients and CS heterogeneity is subject for elucidation taking into account data on mesenchymal cells’ role in differentiation control during development and organogenesis ex vivo.

Among the putative paracrine mediators of this process, recent data indicate a role of several growth factors and morphogenic proteins listed in Table 2. Most of them have a pleiotropic array of functions in development, healing, and regeneration, yet we have chosen to pay specific attention only to proteins actively produced by MSC and upregulated in them in case of integration to scaffolds or tissue-like constructs.

## 7. Concluding Remarks

Present paper reflects our vision on how stem cell delivery might be improved based on knowledge of human development, system biology, and, in particular, the limitations of existing cell therapy approaches. 

To our knowledge, regeneration after injury has not been previously compared to the recovery of an ecosystem structure. Terminology of ecological and evolutionary biology has been extensively used in cancer [112,113,114] and stem cell [115] biology, so we consider our approach reasonable to explain the importance of putative feeders formed in early stages of regeneration for its outcome.

In our view, the participation and recruitment of MSCs during the formation of feeders has a physiological rationale. Our point is supported by a “mapping” role of mesenchymal cells in development and by prominent examples suggesting that adult MSCs retain this ability even ex vivo, driving functional organoid formations and vascularization in a dish. The concept of feeders as a base layer for successful regeneration might be a subject for discussion, yet we suggest it as a summary of our knowledge about regeneration and data from other groups.

This concept overall shifts our understanding of MSC-based CS as a delivery platform for regenerative medicine. We believe that CS is not only a means of “cells + ECM” deployment but may present a unique object for the study of mammalian regeneration, organoid assembly, or tissue modeling. MSCs within CS may retain or reproduce certain traits of mesenchymal cells in development to orchestrate regeneration. Origins and consequences of heterogeneity that we observed in CS, as well as its influence on other cells (including tissue-specific progenitors and terminally differentiated cells), is a subject for further investigation. 

## Figures and Tables

**Table 1:** Biological properties of mesenchymal stromal cells (MSCs) that contribute to their potential as participants of feeder formation.

MSC Property/Function	Reference
Ubiquitous location in tissues and organs	[40,41]
Multipotency and high proliferative potential	[42,43]
Survival under stress conditions and inflammation	[44,45]
Immunomodulation and reduction of inflammation	[29,46,47]
Active pleiotropic secretome comprising of ECM components, growth factors, cytokines, and extracellular vesicles	[31,32,35]
Ability to support and regulate other cell types in vivo	[30,48]

**Table 2:** Paracrine factors produced by MSCs that may play a role in their self-organization and/or their guiding role in regeneration

Growth Factor/Cytokine and its Function	Function and Role in Organized Regeneration	Reference(s)
Transforming growth factor β	Control of fibrosis and morphogenesis, attenuation of immune response and cell survival under stress, activation of ECM production in various cell types	[90,91]
Bone morphogenetic protein family	Resident stem cell activation and control, regeneration and turnover of osseous tissues, control of anterior/posterior axis	[92,93,94]
Platelet-derived growth factors	Potent mitogens in cells of mesenchymal origin, chemoattractant for stromal cells, mediator of wound healing and resident MSC activation	[95,96]
Follistatin	Specific inhibitor of activin A and potent cell cycle controller; plays a crucial role in “shape control” of muscle and skin preventing excessive growth; known to be a part of “missing tissue” sensory system activating regenerative program	[19,97]
Fibroblast growth factors	"Promiscuous" growth factors involved in development, angiogenesis, nerve growth, stromal cell proliferation, and fibrosis after damage, as well as ECM deposition and remodeling	[98,99]
Vascular endothelial growth factors	Control of angiogenesis, cell survival under stress, and pro-inflammatory effects in vascular cells; potent mitogen for MSC and resident stem cells as well as a crucial player in stem cell activation	[100,101,102]
Hepatocyte growth factor	Also known as “scattering factor,” it controls cell assembly and scattering as well as fibrosis and angiogenesis in damaged tissue; known to have anti-inflammatory effect in endothelial and stromal cells	[103,104]
Urokinase plasminogen activator	Potent proteolytic activator of numerous growth factor precursors with pleiotropic effects on ECM remodeling, cell migration, blood vessel and nerve growth	[105,106,107,108]
Wnt-family ligands	De/differentiation control and activation of resident stromal or progenitor cells, as well as potent mitogen and activator of proliferation	[109,110,111]

## Supplementary Materials

Supplementary materials can be found at http://www.mdpi.com/1422-0067/20/4/823/s1.
