# Supplementary material for: Unveiling Mesenchymal Stromal Cells’ Organizing Function in Regeneration
Source: Int J Mol Sci. 2019 Feb 14;20(4):823. doi: 10.3390/ijms20040823 (PMC6413004; doi:10.3390/ijms20040823)
Supplement: Supplementary file 1 [file ijms-20-00823-s001.zip › ijms-436412 supplementary/ijms-436412-supplementary.pdf]

## Supplementary materials and methods

### Cell culture

Human adipose tissue-derived MSC cell line “ASC52telo” (ATCC® SCRC-4000) was cultured in Advance Stem medium (HyClone) containing 10% Advance Stem Supplement (HyClone), 100 units/ml penicillin and 100 units/ml streptomycin with medium change every 3 days. When 75% confluent was reached, cells were passaged using Versene solution and HyQTase (HyClone) solution at a ratio of 1: 4.

### Assembly of cell sheets

To assemble cell sheets MSC were seeded at a high of 15.000 cells/cm2 in 12-well culture plates or in 60mm dishes to form a monolayer control (all materials from Corning). Cells first need to achieve a sufficiently high culture density to assemble the CS which took 7-8 days and at days 12-14 “hills” and “valleys” were formed. After day 10 we changed the medium every 2 days.

### Time-lapse photography

Long-term observation of the CS assembly was carried out using a Nikon ECLIPSE Ti microscope that allows monitoring of live cells in a culture gas mixture, temperature and humidity conditions suitable for long-term existence and growth. Shooting was carried out for 12-16 days, with a 40 minutes frequency. Programmable shooting control in NIS Nikon software was used and obtained videos were edited using Fuji plugin collection for NIH ImageJ.

### Laser microdissection

The laser microdissection was used to separate the “hills” and “valleys” cell subpopulations. For laser microdissection was used Leica laser microdissection system LMD 6000. In our case, we used the following general protocol. MSC was seeded on special microdissection dishes (WillCo - Dish FWST-5030) with a polyethylene bottom. After the assembly of CS, the cells was fixed in 70% ethanol. Then microdissection of the marked “hills” areas was made, and picked out areas with cells on them fall under the action of gravity into the collectors (see figure S1). After that samples was lysed and further qPCR-RT was carried out with them.

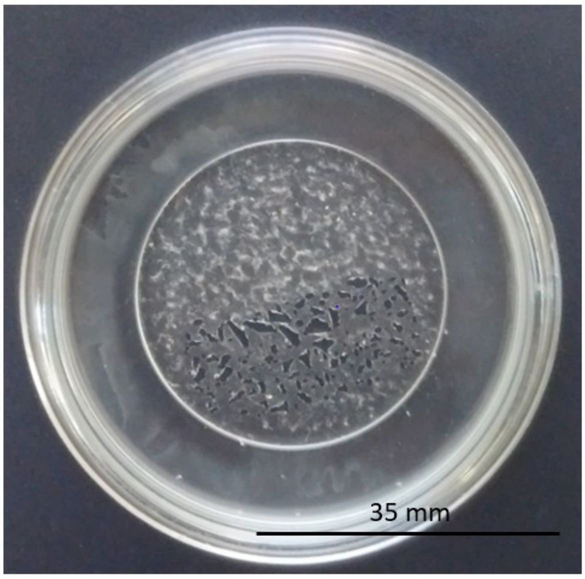


**Figure S1.** Intermediate result of laser microdissection for isolation of "hills". In the upper portion of dish the "hills" are well distinguished while in lower they have already been dissected for mRNA isolation.

**Real-time quantitative RT-PCR**

Microdissection samples after lysis were used for sequential isolation of RNA and reverse transcription reaction and qPCR. Primers were selected using the tool via OligoArchitect (http://www.oligoarchitect.com/), oligonucleotide primer sequences used are listed in Table 1. *RPLA13A* was used as housekeeping gene for normalization. (n=3 experiments in triplicate samples)

**Table S1. Used oligonucleotide sequences of primers (to human genes)**

| Gene | Direct primer 5 '→ 3' | Reverse primer5 '→ 3' |
|---|---|---|
| *RPLA13A* | CTTTCCTCCGCAAGCGGAT | CCACCATCCGCTTTTTCTT |
| *NANOG* | CCTGTGATTTGTGGGCCTG | GACAGTCTCCGTGTGAGGCAT |

**MSC direct staining on a dish**

To visualize the process of CS assembling and associated MSC "hills" self-organization, direct staining of cell culture method was used. CS or monolayer cell culture at certain period of cultivation was fixed with 10% neutral formalin for 10 minutes, then permeabilized by treatment with 70% isopropyl alcohol, then washed with distilled water and stained with hematoxylin for 30 seconds, and then gently rinsed with water for 3 minutes.

**Immunocytofluorescence**

Cell sheets in cell culture dishes were fixed with 4% neutral PBS-formalin for 7 min, washed with PBS 3 times and incubated with 10% donkey serum during 1 hour. Then incubated with primary antibodies to ED A-fibronectin (Abcam, ab6328, USA) and laminin (Abcam, ab11572, USA) during 1 hour, washed with PBS 3 times. Secondary antibodies, conjugated with fluorescent dye were added for 1 hour and washed with PBS 3 times. Cell nuclei were stained with DAPI solution according to the manufacturer's protocol (Sigma-Aldrich, USA). Results were visualized on Leica DM6000B fluorescent microscope equipped with a DFC 360FX camera (Leica Microsystems GmbH, Germany).
